# Bilateral Cerebral Hypoperfusion in Asymptomatic Unilateral Carotid Artery Stenosis: An Arterial Spin Labeling MRI Study

**DOI:** 10.3390/medicina61050771

**Published:** 2025-04-22

**Authors:** Nikola Dacic, Srdjan Stosic, Olivera Nikolic, Zoran D. Jelicic, Aleksandra Dj Ilic, Mirna N. Radovic, Jelena Ostojic

**Affiliations:** 1 Center for Radiology, University Clinical Center of Vojvodina, 21000 Novi Sad, Serbia; 1238d17@mf.uns.ac.rs (N.D.); srdjan.stosic@mf.uns.ac.rs (S.S.); olivera.nikolic@mf.uns.ac.rs (O.N.); 2 Faculty of Medicine, University of Novi Sad, 21000 Novi Sad, Serbia; aleksandra.dj.ilic@mf.uns.ac.rs; 3 Department of Computing and Control, Faculty of Technical Sciences, University of Novi Sad, 21000 Novi Sad, Serbia; jelicic@uns.ac.rs (Z.D.J.); mirna.kapetina@uns.ac.rs (M.N.R.); 4 Neurology Clinic, University Clinical Center of Vojvodina, 21000 Novi Sad, Serbia

**Keywords:** carotid artery stenosis, cerebral blood flow, arterial spin labeling, magnetic resonance perfusion, hypoperfusion, insular cortex, middle cerebral artery

## Abstract

*Background and Objectives*: Carotid artery stenosis is a significant risk factor for ischemic stroke due to impaired cerebral blood flow (CBF). Even asymptomatic unilateral stenosis can induce subclinical cerebrovascular changes, potentially affecting both hemispheres through collateral circulation. This study aimed to systematically assess cerebral perfusion in asymptomatic individuals with unilateral carotid artery stenosis by comparing ipsilateral and contralateral hemispheres with healthy controls, challenging the assumption that the contralateral hemisphere remains unaffected. *Materials and Methods:* This cross-sectional study included 114 participants, comprising 54 asymptomatic individuals (mean age 65.5) with significant unilateral carotid stenosis and 60 age-matched controls (mean age 64.8). Cerebral perfusion was assessed using 1.5T Magnetic Resonance Imaging (MRI) with pseudo-continuous arterial spin labeling (pCASL). CBF was measured bilaterally in four predefined middle cerebral artery (MCA) regions: precentral gyrus, lentiform nucleus, insular cortex, and temporal cortex. Statistical analyses included multivariate analysis of variance (MANOVA), analysis of variance (ANOVA), paired *t*-tests, and discriminant analysis (DA). *Results:* Significant bilateral reductions in CBF were observed in individuals with carotid stenosis compared to controls (MANOVA and ANOVA, *p* < 0.001). The greatest perfusion deficit was in the ipsilateral insular cortex (49.88 ± 10.83 mL/100 g/min), followed by intermediate contralateral perfusion (51.49 ± 8.86 mL/100 g/min) and higher control values (58.78 ± 10.44 mL/100 g/min). DA indicated the insular cortex as the region with the highest discriminative contribution (64.7%). *Conclusions*: Unilateral carotid artery stenosis in asymptomatic individuals is associated with significant bilateral cerebral hypoperfusion, suggesting widespread hemodynamic effects. Pronounced perfusion deficits in the insular cortex underline its vulnerability. The observed contralateral perfusion reductions challenge the traditional use of the contralateral hemisphere as a reference standard, underscoring the need for comprehensive perfusion assessment in carotid artery disease.

## 1. Introduction

Carotid artery stenosis is a significant risk factor for ischemic stroke and transient ischemic attacks (TIAs), as it restricts blood flow to the brain [1]. Atherosclerotic narrowing of the internal carotid artery (ICA) can reduce cerebral blood flow (CBF), particularly in the middle cerebral artery (MCA) territory, potentially contributing to hypoperfusion and increased susceptibility to ischemic injury [2]. High-grade carotid stenosis often leads to altered cerebral hemodynamics, including reduced perfusion in the ipsilateral hemisphere and potential compensatory mechanisms through the anterior communicating artery (ACoA) and posterior communicating artery (PCoA) [3,4]. The degree of this compensation varies among individuals, influencing their susceptibility to ischemic events [5]. The MCA territory is highly relevant in cerebrovascular research due to its vascularization of both cortical and subcortical structures, which are functionally critical and frequently affected in cerebrovascular disease [6]. Given that the MCA is the most commonly affected artery in ischemic stroke, investigating perfusion changes in this region is crucial for understanding the impact of carotid stenosis [7]. Cortical and subcortical structures within the MCA territory are particularly susceptible to hemodynamic compromise in carotid artery disease, although recent findings also suggest a degree of functional resilience despite chronic hypoperfusion [8,9]. Arterial Spin Labeling (ASL) magnetic resonance imaging (MRI) is a non-invasive imaging technique that enables quantitative assessment of cerebral perfusion without the need for contrast agents [10]. It measures CBF by magnetically labeling arterial blood before it enters the capillary network, allowing for a direct evaluation of brain perfusion under physiological conditions. ASL has been widely used to assess CBF in various cerebrovascular conditions, including carotid stenosis, providing valuable insights into regional perfusion deficits and aiding in the evaluation of disease severity and progression [6,11]. In our study, we employed long-label pseudo-continuous arterial spin labeling (pCASL), which has demonstrated good test–retest reliability and reproducibility in anterior brain regions in elderly populations [12]. Previous studies have established that patients with significant carotid stenosis exhibit reduced CBF in the ipsilateral hemisphere, confirming the hemodynamic impact of arterial narrowing [13,14,15]. The use of ASL in this context has also been validated against gold-standard methods such as positron emission tomography using radiolabeled water as a tracer ([^15^O]H_2_O PET) [16], reinforcing its utility in detecting hemodynamic compromise in cerebrovascular disease. Several studies have reported varying findings regarding cerebral perfusion in patients with high-grade ICA stenosis. Some studies have demonstrated reduced contralateral CBF [15,17,18], while others have reported no significant change [19,20]. Conversely, increased contralateral perfusion has also been described, suggesting a potential compensatory mechanism [21,22]. These inconsistencies in reported perfusion patterns underscore the lack of consensus regarding the impact of ICA stenosis on the contralateral hemisphere, highlighting the need for further investigation to establish whether contralateral perfusion is reduced, unchanged, or compensatorily increased in this patient population. This need is particularly relevant in asymptomatic patients, in whom subclinical hemodynamic disturbances may go undetected, despite the absence of overt neurological symptoms [23]. Emerging evidence suggests that asymptomatic carotid stenosis may not be entirely benign and could be associated with covert ischemic changes, cognitive impairment, and increased long-term risk [24]. Additionally, this study offers a more detailed regional analysis of perfusion changes within specific brain structures in the MCA territory by examining four distinct locations, thereby providing further insights into the spatial heterogeneity of perfusion dynamics. Given that different brain regions have distinct vulnerabilities to hypoperfusion, a region-specific investigation is necessary to understand the full extent of hemodynamic alterations in unilateral carotid stenosis.

This study aims to systematically evaluate cerebral perfusion changes in asymptomatic patients with unilateral carotid artery stenosis by comparing CBF values in the ipsilateral and contralateral hemispheres, as well as in corresponding regions of healthy individuals. To assess regional variation in perfusion, analysis will focus on four representative brain structures within the MCA territory. By providing a region-specific assessment of CBF alterations, this study seeks to expand upon prior research and improve our understanding of cerebrovascular adaptation mechanisms in the context of unilateral carotid stenosis.

## 2. Materials and Methods

### 2.1. Study Population and Clinical Characteristics

A total of 114 individuals were enrolled in the study, consisting of 54 patients with unilateral carotid artery stenosis greater than 75% (age range: 55–80 years; mean age: 65.5) and 60 age-matched controls (mean age: 64.8) [25]. Of the 54 patients, 30 exhibited no abnormalities on conventional magnetic resonance imaging (MRI), whereas 24 demonstrated chronic microvascular white matter lesions consistent with Fazekas grade 1 or 2. Across all participants, gray matter was structurally preserved, with no detectable lesions or evidence of cortical pathology. All subjects were evaluated at the Vascular Surgery and Neurology Clinic, where they underwent Doppler ultrasonography, computed tomography angiography (CTA), and/or magnetic resonance angiography (MRA). Based on these imaging modalities, patients with significant unilateral carotid artery stenosis were identified and selected for inclusion. Individuals with extensive ischemic damage or any other form of brain pathology, aside from chronic microvascular changes, were excluded. The control cohort consisted of participants without evidence of carotid artery stenosis or brain lesions; however, some exhibited common vascular risk factors, such as hypertension (HTN) or diabetes mellitus (DM), in the absence of structural brain abnormalities. Demographic and clinical characteristics of all participants are summarized in Table 1.

There were no statistically significant differences in age or sex distribution between the stenosis and control groups (*p* > 0.05). In contrast, significant group differences were observed in lipid profile parameters, with a higher prevalence of hyperlipoproteinemia (HLP) among patients with carotid stenosis. Moreover, the proportion of smokers was significantly greater in the stenosis group compared to the controls.

### 2.2. MRI Acquisition Protocol

MRI, including ASL, was performed on a 1.5 T Signa MRI scanner (General Electric Medical Systems, Chicago, IL, USA) using an eight-channel head coil. The MRI protocol included a sagittal T1-weighted spin-echo (T1-SE) sequence with a repetition time (TR) of 440 ms and an echo time (TE) of 3.8 ms, a slice thickness of 5 mm, and an acquisition time of 3 min. Axial T2-weighted (T2W) fast spin echo (FSE) sequences were acquired with a TR/TE of 4120/92 ms, a slice thickness of 5 mm, and an acquisition time of 2 min and 49 s. Axial fluid-attenuated inversion recovery (FLAIR) sequences were obtained with a TR/TE of 8802/144 ms, a slice thickness of 5 mm, and an acquisition time of 4 min and 51 s. Diffusion-weighted imaging (DWI) was performed with a TR/TE of 8000/100 ms, a slice thickness of 5 mm, and b-values of 0 and 1000 s/mm^2^, with an acquisition time of 2 min and 8 s. Three-dimensional susceptibility-weighted angiography (3D SWAN) was obtained with a TR/TE of 78.3/48 ms, a slice thickness of 1.5 mm, and an acquisition time of 5 min and 17 s. A 3D BRAVO T1 sequence was acquired with a TR/TE of 8.9/3.5 ms, a slice thickness of 1 mm, and an acquisition time of 3 min and 39 s. Angiographic imaging included three-dimensional time-of-flight (3D TOF) magnetic resonance angiography (MRA) performed separately for both the brain and the neck, each acquired with a TR/TE of 28/3.4 ms and an acquisition time of 5 min and 27 s.

### 2.3. Arterial Spin Labeling Acquisition and Quantification

A long-label 3D pseudo-continuous arterial spin labeling (pCASL) sequence was performed with a TR/TE of 4650/10.5 ms, a slice thickness of 4 mm, a labeling duration (LD) of 2000 ms, a post-labeling delay (PLD) of 2000 ms, a flip angle of 15°, and a labeling plane positioned 4 cm below the circle of Willis. The readout was performed using a three-dimensional Gradient and Spin Echo (3D-GRASE) acquisition, which provides an improved signal-to-noise ratio (SNR) and reduces sensitivity to transit time artifacts. To minimize static tissue signal and enhance perfusion contrast, two background suppression pulses were applied. Motion correction algorithms were implemented to account for potential patient movement, and three signal averages (NSA—number of signal averages) were used to further optimize SNR and the reliability of cerebral blood flow (CBF) quantification. The acquisition time for this sequence was 4 min and 12 s. The pCASL sequence was specifically optimized for assessing CBF by employing a long LD and an extended PLD, ensuring robust perfusion quantification, particularly in regions affected by delayed arterial transit time. The total scan duration for the entire MRI protocol was 36 min and 50 s. Post-processing and analysis were performed using the Advantage Workstation, software version 4.7 (GE Medical Systems/Healthcare, Waukesha, WI, USA). To quantify cortical perfusion in the middle cerebral artery (MCA) territory, four regions of interest (ROIs) were manually delineated bilaterally in the precentral gyrus cortex, insular cortex, lentiform nucleus, and temporal cortex (temporopolar region).

**Figure 1:**
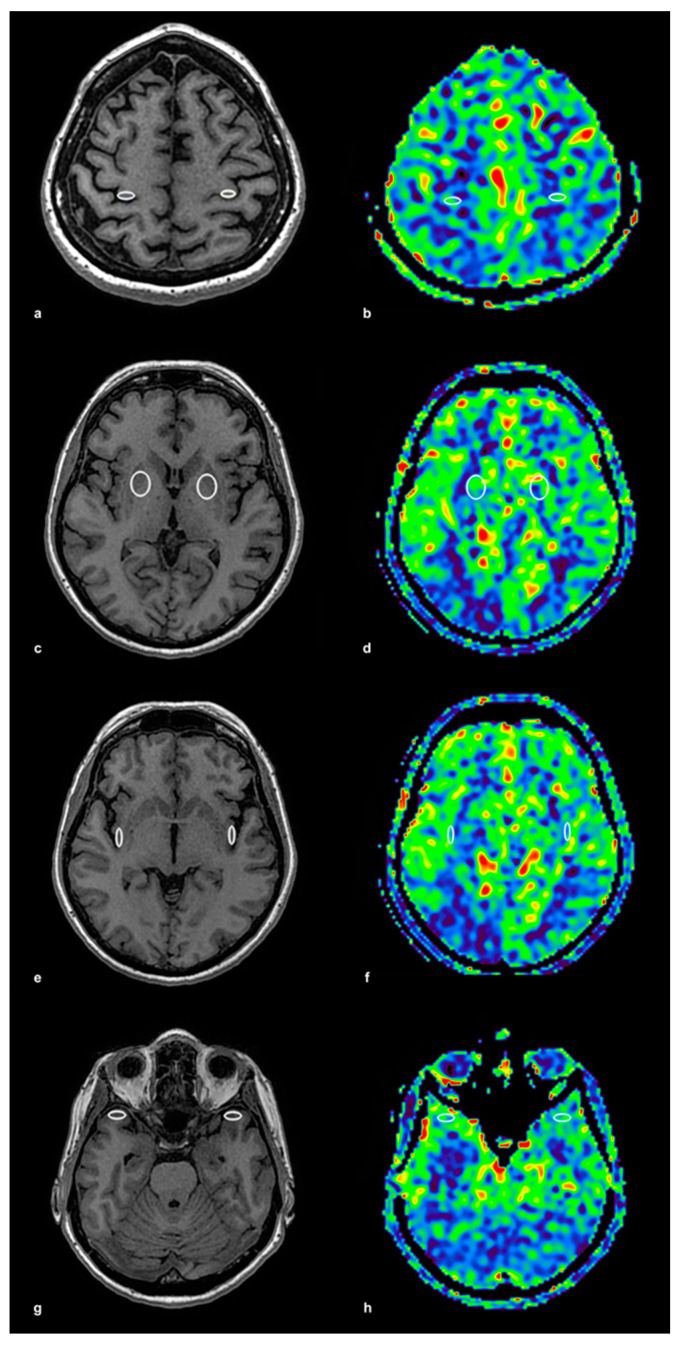
ROI Placement and ASL Perfusion Map in a Control Subject. This figure illustrates the placement of regions of interest (ROIs) on T1-weighted anatomical images and their corresponding arterial spin labeling (ASL) perfusion maps in a control subject. (**a**) T1-weighted image with ROI placement in the precentral gyrus (frontal cortex) bilaterally, ensuring precise localization in the gray matter, and (**b**) the corresponding ASL perfusion map with identically positioned ROIs for cerebral blood flow (CBF) quantification. (**c**) T1-weighted image with ROI placement in the lentiform nucleus bilaterally, and (**d**) the corresponding ASL perfusion map. (**e**) T1-weighted image with ROI placement in the insular cortex bilaterally, and (**f**) the corresponding ASL perfusion map. (**g**) T1-weighted image with ROI placement in the temporal cortex bilaterally, and (**h**) the corresponding ASL perfusion map. ROI sizes ranged from 15 to 40 mm^2^, with elliptical ROIs used in thinner cortical regions and circular ROIs in larger areas. The colored ASL perfusion maps (**b**,**d**,**f**,**h**) represent quantitatively measured cerebral blood flow (CBF), with warmer colors (red, yellow) corresponding to higher CBF values and cooler colors (green, blue) indicating lower values.

**Figure 2:**
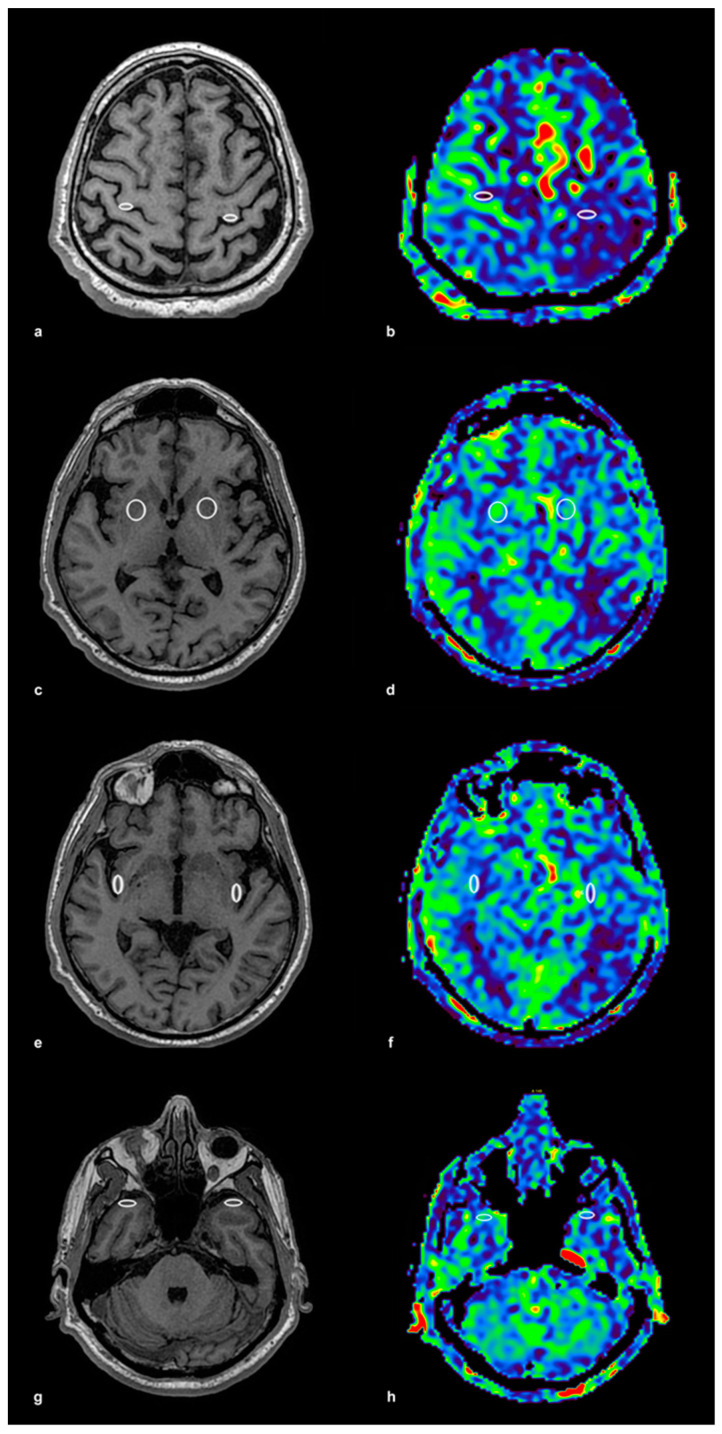
ROI Placement and ASL Perfusion Map in a Patient with Carotid Stenosis. This figure presents ROI placement and ASL perfusion maps in a patient with unilateral carotid stenosis, following the same methodology as in Figure 1. (**a**) T1-weighted image with ROI placement in the precentral gyrus (frontal cortex) bilaterally, and (**b**) the corresponding ASL perfusion map with the same ROIs transferred for CBF quantification. (**c**) T1-weighted image with ROI placement in the lentiform nucleus bilaterally, and (**d**) the corresponding ASL perfusion map. (**e**) T1-weighted image with ROI placement in the insular cortex bilaterally, and (**f**) the corresponding ASL perfusion map. (**g**) T1-weighted image with ROI placement in the temporal cortex bilaterally, and (**h**) the corresponding ASL perfusion map. ROI sizes (15–40 mm^2^) were adjusted according to the anatomical region, with elliptical ROIs applied in thinner cortical regions and circular ROIs in larger areas. The colored ASL perfusion maps (**b**,**d**,**f**,**h**) represent quantitatively measured cerebral blood flow (CBF), with warmer colors (red, yellow) corresponding to higher CBF values and cooler colors (green, blue) indicating lower values.

The ROIs were initially placed on high-resolution 3D T1-weighted anatomical images, ensuring precise localization within the gray matter, and then automatically transferred to the corresponding ASL perfusion maps acquired with identical geometric parameters. This method ensured accurate spatial alignment between anatomical and perfusion imaging. The ROI placement procedure is illustrated in Figure 1, using a control subject to demonstrate anatomical accuracy and spatial consistency. In panel (a), bilateral ROIs were positioned within the precentral gyrus on T1-weighted anatomical images, while panel (b) shows the corresponding ASL perfusion map with these ROIs overlaid for CBF quantification. Panels (c,d) depict the lentiform nucleus, again with paired anatomical and perfusion images. The same approach was applied to the insular cortex (e,f) and the temporal cortex (g,h), ensuring consistent methodology across all examined brain regions. To illustrate the same ROI protocol in a patient with significant unilateral carotid artery stenosis, Figure 2 presents corresponding anatomical and perfusion images. As in the control example, ROIs were bilaterally placed in the precentral gyrus, lentiform nucleus, insular cortex, and temporal cortex. Panel (a) shows ROI localization on T1-weighted images for the precentral gyrus, and panel (b) displays the matching ASL perfusion map. Panels (c,d) represent the lentiform nucleus, (e,f) the insular cortex, and (g) and (h) the temporal cortex, with anatomical and perfusion images paired in each case. This consistent ROI placement ensured reproducibility of the analysis and facilitated accurate intergroup comparisons of regional CBF values. ROI sizes ranged from 15 to 40 mm^2^, depending on the anatomical region, individual morphological variation, and the presence of age-related brain atrophy. Elliptical ROIs were applied to thinner cortical structures, such as the insular and precentral cortices, while circular ROIs were used in larger subcortical areas, including the lentiform nucleus and temporal cortex. This adjustment provided robust and anatomically valid perfusion estimates across the study population.

### 2.4. Statistical Analysis

All statistical analyses were conducted using a uniform significance threshold of *p* < 0.05 to assess the significance of observed differences in cerebral blood flow (CBF) across brain regions. Initially, we compared bilateral CBF values (left vs. right hemisphere) within the control group. As no significant differences were observed in any of the examined brain regions, the measurements from both hemispheres in the 60 control participants were combined into a single control group for further analysis. Based on this dataset, we subsequently performed a multivariate analysis of variance (MANOVA) to examine global differences among the three groups: the control group, the hemisphere ipsilateral to the stenotic carotid artery, and the contralateral hemisphere. Following significant MANOVA results, we performed follow-up univariate analyses, including one-way analysis of variance (ANOVA) and Student’s *t*-tests, to identify region-specific differences in CBF between groups. In addition, discriminant analysis (DA) was used to determine the relative contribution of each brain region to group classification. The analysis yielded coefficients that identified the brain regions with the highest discriminatory power in separating the control group, the ipsilateral hemisphere, and the contralateral hemisphere.

## 3. Results

The distribution of CBF values across all examined brain regions was approximately normal, as indicated by descriptive statistics and dispersion parameters (Table 2, Table 3 and Table 4). Initially, we compared CBF values between the left and right hemispheres within the control group at all examined locations. As there were no significant differences at any of the examined locations, measurements from the 60 control subjects were combined into a single group, resulting in a total of 120 measurements per region. MANOVA demonstrated significant differences in CBF between the ipsilateral, contralateral, and control groups across all examined brain regions (*p* < 0.001). While ANOVA confirmed the presence of significant differences (*p* < 0.05) at all examined locations, it did not specify which groups differed from one another. To precisely determine these differences, pairwise comparisons were performed using two-tailed *t*-tests for each brain region, with results summarized in Table 5. Only locations exhibiting statistically significant intergroup differences (*p* < 0.05) are reported. The *t*-test revealed significant differences in CBF among the three groups, control (Group 1), ipsilateral (Group 2), and contralateral (Group 3), across multiple brain regions. In the lentiform nucleus, CBF was significantly lower in the ipsilateral group compared to the control group (t = 3.230, *p* = 0.001) and also lower in the contralateral group compared to the control group (t = 2.383, *p* = 0.018). In the frontal cortex-precentral gyrus, CBF was significantly lower in the ipsilateral group compared to the control group (t = 4.117, *p* < 0.001) and also lower in the contralateral group compared to the ipsilateral group (t = 3.221, *p* = 0.002). In the insular cortex, CBF was significantly lower in the ipsilateral group compared to the control group (t = 5.147, *p* < 0.001), and also lower in the contralateral group compared to the control group (t = 4.458, *p* < 0.001). In the temporal cortex, CBF was significantly lower in the ipsilateral group compared to the control group (t = 4.039, *p* < 0.001), and also lower in the contralateral group compared to the ipsilateral group (t = 2.471, *p* = 0.015).

**Figure 3:**
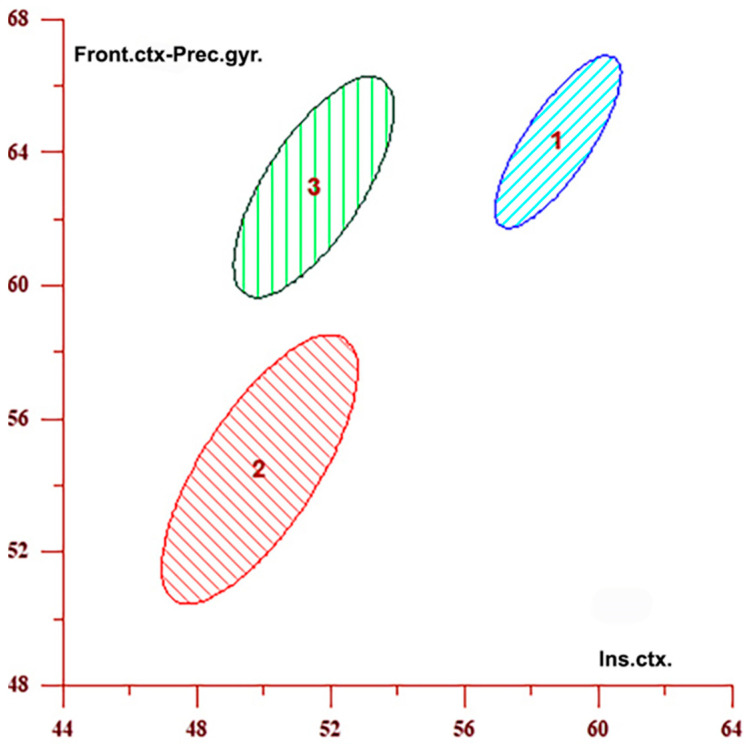
The graph displays confidence interval ellipses representing CBF values in the regions that contribute the most to the differentiation between the ipsilateral (2) and contralateral (3) hemispheres in individuals with unilateral stenosis, as well as the control group (1), based on Table 6. These values were obtained in the insular cortex and the frontal cortex–precentral gyrus, which are the first two variables listed in Table 6. The graph shows that CBF values are highest in the control group (1), intermediate in the contralateral hemisphere (3), and lowest in the ipsilateral hemisphere (2) in individuals with unilateral stenosis, and that these groups are clearly separated.

**Figure 4:**
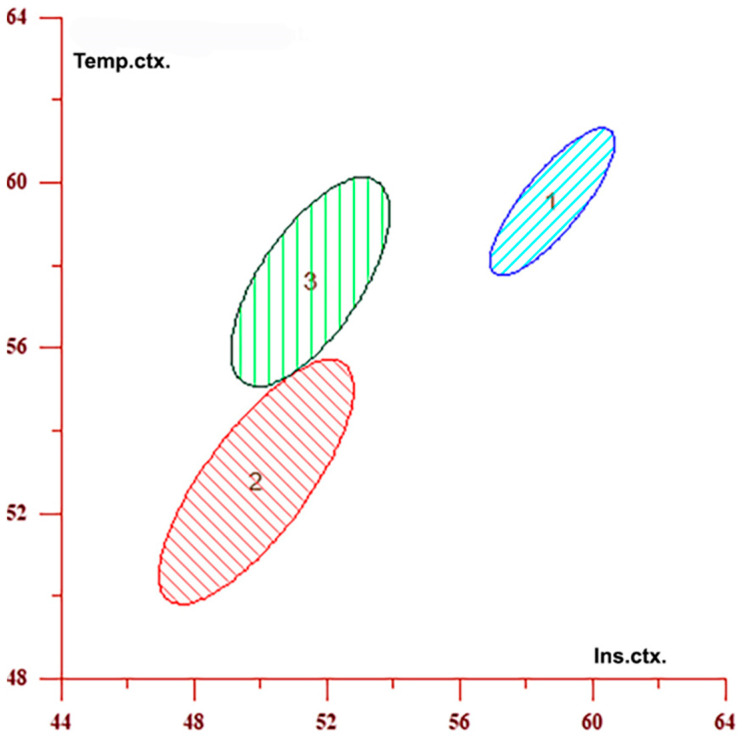
The graph displays confidence interval ellipses representing CBF values in the regions that contribute the most to the differentiation between the ipsilateral (2) and contralateral (3) hemispheres in individuals with unilateral stenosis, as well as the control group (1), based on Table 6. These values were obtained in the insular and temporal cortex, which are the first and third variables listed in Table 6. The graph shows that CBF values are highest in the control group (1), intermediate in the contralateral hemisphere (3), and lowest in the ipsilateral hemisphere (2) in individuals with unilateral stenosis. Furthermore, groups 2 and 3 (ipsilateral and contralateral hemispheres) are closer to each other, whereas the control group is clearly distinct from both.

Discriminant analysis (Table 6) identified distinct perfusion profiles for each group and quantified the contribution of individual brain regions to these patterns. The insular cortex contributed the most to group differentiation, with a 64.684% contribution, followed by the frontal cortex (18.216%), the temporal cortex (10.781%), and the lentiform nucleus (6.320%). Across all regions, CBF values were highest in the control group, lowest in the ipsilateral hemisphere, and intermediate in the contralateral hemisphere. The three regions with the highest discriminative power are presented both numerically and graphically. These relationships are further illustrated in Figure 3 and Figure 4, which display confidence interval ellipses derived from DA for the brain regions with the highest contribution to group differentiation. Figure 3 shows CBF distributions in the insular cortex and the frontal cortex–precentral gyrus, whereas Figure 4 illustrates distributions in the insular and temporal cortex. In both graphs, CBF values are highest in the control group (Group 1), intermediate in the contralateral hemisphere (Group 3), and lowest in the ipsilateral hemisphere (Group 2), consistent with the numerical data presented in Table 6. The visual separation between the ellipses reflects significant differences in CBF among all three groups. Notably, CBF values in the contralateral hemisphere (Group 3) also differ from those in the control group (Group 1), despite the absence of direct vascular compromise.

## 4. Discussion

This study demonstrated bilateral cerebral hypoperfusion in asymptomatic patients with unilateral carotid artery stenosis >75%, using ASL MRI. CBF was significantly reduced not only in the ipsilateral hemisphere but also in the contralateral one, suggesting that high-grade stenosis may induce widespread hemodynamic compromise even in the absence of clinical symptoms. These findings suggest that CBF alterations extend beyond the directly affected hemisphere, implicating systemic vascular mechanisms that influence global perfusion. Importantly, all patients included in this study were asymptomatic, allowing us to examine subclinical cerebrovascular alterations associated with high-grade carotid stenosis in the absence of overt neurological symptoms. While carotid stenosis primarily compromises the ipsilateral hemisphere, our results demonstrate that contralateral perfusion is also significantly reduced. This finding can be attributed to hemodynamic mechanisms directly linked to carotid stenosis, including reduced cerebral perfusion pressure [13], impaired collateral circulation [26], and compensatory vasodilation [1] that may be insufficient to maintain adequate CBF. Reduced perfusion in major watershed territories has been reported in patients with asymptomatic ICA stenosis, supporting our observations of bilateral hypoperfusion [22,27]. In cases of severe carotid stenosis, diminished perfusion pressure across the stenotic segment leads to a decrease in downstream CBF [14]. The ability of collateral pathways, such as the circle of Willis, to compensate for reduced flow is a key determinant of the extent of perfusion impairment. If these collateral routes are insufficient, both the ipsilateral and contralateral hemispheres may experience hypoperfusion [4,28]. While cerebral autoregulatory mechanisms, including the vasodilation of distal resistance arteries, may become exhausted in patients with carotid stenosis [29], studies in healthy elderly individuals have shown that dynamic cerebral autoregulation and cerebrovascular CO_2_ reactivity can be preserved despite age-related changes in cerebral hemodynamics [30]. Such variability across individuals underscores the need for the direct assessment of autoregulatory function in carotid disease. These perfusion deficits have been linked to increased risks of ischemic events and cognitive decline [31]. The reduction in CBF observed in both hemispheres due to carotid stenosis or occlusion may be associated with an impaired cerebrovascular reserve (CVR), suggesting that compensatory mechanisms such as collateral circulation and vasodilation could be insufficient to maintain adequate perfusion levels, although direct assessment of CVR is required for confirmation [32,33]. Furthermore, increased arterial stiffness associated with aging may reduce cerebral autoregulatory capacity and exacerbate perfusion deficits, highlighting the multifactorial nature of cerebrovascular compromise in carotid artery disease [34]. Cerebral autoregulatory capacity refers to the brain’s ability to maintain stable blood flow despite fluctuations in systemic blood pressure, primarily through the dilation and constriction of cerebral arterioles. Cerebrovascular compromise denotes any condition that impairs blood flow within the brain’s vasculature, potentially leading to ischemia or other neurological deficits. CVR, which represents the brain’s ability to augment blood flow in response to metabolic demands, is a critical determinant of stroke risk [32]. Patients with severe carotid stenosis often exhibit an exhausted CVR, increasing their vulnerability to ischemic events. Our findings align with the concept of cerebral hemodynamic impairment, in which CBF is critically reduced, necessitating compensatory mechanisms such as increased oxygen extraction fraction (OEF) to maintain metabolic demand [13]. Although we did not directly assess CVR, the observed ASL-derived CBF reductions may reflect impaired vasodilatory capacity. Since our study was cross-sectional, we were unable to evaluate longitudinal outcomes or prognostic implications of reduced CBF. Further studies using functional testing, such as CO_2_ reactivity or acetazolamide challenge, are needed to confirm these findings. A significant reduction in contralateral perfusion compared to control subjects was observed, aligning with studies that report decreased contralateral CBF in patients with carotid stenosis [15,17,18]. Notably, Li et al. specifically examined patterns of acute contralateral ischemic stroke in patients with unilateral extracranial internal carotid artery stenosis and found that hemodynamic impairment on the contralateral side could be attributed to reduced perfusion pressure and collateral flow inefficiencies [17]. Their findings suggest that in certain cases, contralateral ischemic events may result from systemic vascular disturbances rather than solely embolic mechanisms. These observations are consistent with our findings, where contralateral hypoperfusion was identified even in asymptomatic patients, underscoring the potential for subclinical cerebrovascular dysfunction. Such results challenge the assumption commonly held in earlier research, where the contralateral hemisphere was used as a control in cerebrovascular studies. Recent findings from resting-state fMRI also question the reliability of using the contralateral hemisphere as a reference in asymptomatic carotid stenosis, showing altered cerebral hemodynamics that are not limited to the ipsilateral side, even in the absence of clinical symptoms [2]. Complementing these observations, an ASL study by Luijten et al. demonstrated that regional CBF is significantly reduced in areas affected by vascular lesions, reinforcing the notion that contralateral hemispheric regions may also be compromised and should not always be assumed as unaffected controls in cerebrovascular research [35]. However, given our findings and those of other studies, the contralateral hemisphere may not always serve as a reliable reference point for comparison due to the presence of systemic and collateral perfusion changes. Accurate interpretation of cerebral hemodynamics in such patients may therefore require the use of absolute perfusion metrics rather than relative interhemispheric comparisons. These considerations are supported by recent ASL findings from Lu et al., who demonstrated that perfusion in the contralateral hemisphere is influenced by systemic hemodynamic factors and collateral flow dynamics in patients with carotid artery stenosis, underscoring the complexity and adaptability of cerebral perfusion mechanisms [36]. In patients with carotid artery stenosis, higher contralateral CBF has been linked to better cerebrovascular compensation and functional recovery [18]. Their study demonstrated that individuals with preserved contralateral perfusion exhibited improved outcomes at 90 days, suggesting that contralateral CBF may reflect the brain’s adaptive response to compromised ipsilateral flow. This observation reinforces the view that contralateral perfusion is not merely a passive reference but an active determinant of compensation and prognosis. Higher contralateral CBF has been associated with better cerebrovascular reserve and functional recovery, suggesting its potential role as a prognostic marker in stroke patients [1,18,37]. However, given our findings of reduced contralateral CBF even in asymptomatic patients, it is possible that lower contralateral perfusion could indicate impaired cerebrovascular reserve, increasing the risk of future ischemic events. Further studies are needed to confirm this potential association and determine its clinical implications. Traditionally, neurological symptoms have been a key determinant in guiding the management of carotid stenosis, with treatment recommendations favoring intervention in symptomatic patients, while management in asymptomatic cases remains more conservative [38]. Yet, our findings call attention to the possibility that asymptomatic carotid stenosis may also harbor significant hemodynamic compromise, which current clinical algorithms might overlook. However, our findings add to a growing body of evidence indicating that even asymptomatic carotid stenosis may be associated with covert cerebrovascular dysfunction, including global reductions in CBF [39]. Previous studies have shown that asymptomatic carotid stenosis is not necessarily benign and may be linked to subclinical ischemic injury, white matter hyperintensities, and cognitive impairment [23,40,41]. The observed bilateral hypoperfusion in our cohort suggests that systemic hemodynamic disturbances can occur even in the absence of apparent clinical events. Regional analysis revealed that the insular cortex provides the greatest contribution to the differentiation between the ipsilateral, contralateral, and control groups in patients with carotid stenosis. The precentral gyrus follows in terms of its contribution to this differentiation, although to a much lesser extent. This suggests that the insula may serve as a sensitive imaging biomarker for subclinical hemodynamic stress in carotid artery disease. This pattern is consistent with prior research indicating that the insula is particularly vulnerable to ischemic events due to its unique vascular supply from the M2 segment of the MCA [42,43]. Given its crucial role in autonomic regulation and cardiovascular control, hypoperfusion in the insular cortex may have significant clinical implications, including an increased risk of arrhythmias and hemodynamic instability [44]. While the precentral gyrus plays a smaller role in distinguishing the groups, reductions in CBF in this region may still have functional relevance, particularly concerning subtle motor impairments that may not be immediately apparent in standard neurological assessments [45]. These findings call for a reassessment of current risk stratification strategies for individuals with asymptomatic carotid stenosis. While current guidelines, such as the European Society for Vascular Surgery (ESVS) 2023 Clinical Practice Guidelines [46], emphasize intervention primarily for patients with high-grade stenosis or microembolic signals detected via transcranial Doppler (TCD), our results, along with previous studies [15,47,48], suggest that CBF assessment could provide additional insight into cerebrovascular risk. Emerging data support the integration of perfusion imaging into clinical decision-making, especially for identifying asymptomatic patients who may benefit from earlier monitoring or intervention. Recent literature also highlights emerging high-risk features that may warrant earlier intervention in asymptomatic patients [49]. Carotid endarterectomy (CEA) and carotid artery stenting (CAS) have been shown to significantly improve ipsilateral CBF by restoring normal hemodynamic flow [50]. However, their impact on contralateral CBF remains inadequately explored. Some studies indicate a potential increase in contralateral CBF following ipsilateral revascularization, but it is unclear whether this represents true normalization or compensatory redistribution [51]. Further research is needed to clarify whether these effects reflect true normalization or ongoing compensatory adaptation. Beyond stroke risk, reduced CBF in patients with carotid stenosis has been associated with cognitive impairment and an increased burden of silent infarcts [21,52]. Chronic cerebral hypoperfusion has been implicated in the pathogenesis of vascular cognitive impairment and dementia, with neuroinflammation playing a central role in this process [53]. Taken together, our results support future investigations that combine perfusion imaging with cognitive and inflammatory biomarkers to explore this multidimensional relationship. Several limitations of this study should be acknowledged. The relatively small sample size may limit the generalizability of our findings to broader populations and warrants validation in larger cohorts. Although ASL was performed on a 1.5 T MRI scanner (General Electric Medical Systems, Chicago, IL, USA) and yielded robust and clinically meaningful CBF measurements, imaging at 3T may offer enhanced sensitivity and spatial resolution, potentially enabling more detailed regional analyses. We did not assess CVR directly using functional tests such as CO_2_ reactivity or acetazolamide challenge, so interpretations related to impaired vasodilatory capacity remain indirect. Additionally, post-treatment perfusion changes following carotid endarterectomy or stenting were not evaluated, making it impossible to draw conclusions about the reversibility of hypoperfusion. The cross-sectional study design limits the ability to determine whether contralateral hypoperfusion in asymptomatic carotid stenosis patients predicts future ischemic events or clinical deterioration. Although ROI placement was anatomically standardized and spatially aligned across participants, subtle variations due to individual cortical morphology cannot be entirely excluded. This potential variability is inherent to manual delineation approaches and may influence the precision of region-specific perfusion estimates. Finally, cognitive function and neuroinflammatory biomarkers were not included in the present study, which limits our ability to explore potential associations between hypoperfusion, neuroinflammation, and cognitive decline. Future studies should aim to address these limitations by including larger patient cohorts, performing longitudinal follow-up, incorporating functional hemodynamic testing, and evaluating the relationship between cerebral perfusion, inflammation, and cognitive outcomes. Additionally, 3T ASL protocols should be utilized where available to enhance spatial resolution and signal-to-noise ratio.

## 5. Conclusions

This study shows that significant unilateral carotid disease in asymptomatic individuals is associated with bilateral reductions in cerebral blood flow, suggesting that its hemodynamic consequences extend beyond the ipsilateral vascular territory. The observed contralateral hypoperfusion indicates that unilateral carotid disease may induce global cerebrovascular compromise, challenging the assumption that the asymptomatic state is benign. Furthermore, the insular cortex was identified as particularly vulnerable to perfusion deficits, underscoring its critical role in autonomic regulation and known susceptibility to ischemia. These findings support the view that cerebral perfusion imaging can uncover subclinical pathology in patients with carotid disease and may offer additional insight into cerebrovascular health beyond traditional imaging and symptom-based assessment, potentially informing earlier risk stratification and management strategies.

## Figures and Tables

**Table 1:** Patient characteristics.

	Significant Stenosis N = 54	Control N = 60	*t*-Test/χ^2^/*p* Value
Age (mean, SD, range)	64.8 (±6.932; 53–80)	65.5 (±6.905; 55–80)	>0.05
Sex			
Male	29 (53.70%)	28 (46.67%)	>0.05
Female	25 (46.30%)	32 (53.33%)	
Comorbidities			
HTN	42 (77.78%)	40 (66.6%)	>0.05
DM	12 (22.2%)	9 (15%)	>0.05
HLP	35 (64.81%)	20 (33.3%)	<0.001
Previous Stroke	0 (0%)	0 (0%)	
Harmful Habits			
Alcohol	6 (11.1%)	5 (8.33%)	>0.05
Tobacco	26 (48.15%)	19 (31.67%)	<0.05

Differences in the presented parameters are shown at a statistical significance level of *p* < 0.05 or *p* < 0.001.

**Table 2:** Descriptive statistics, asymmetry, and kurtosis measures of brain parenchyma CBF in the ACM territory of control subjects, with preserved width of both ACIs (120).

Reg.	Mean	SD	Min	Max	CV	CI		Skew	Kurt	*p*-Value
Lent. nuc.	51.6	9.12	32.4	79.8	17.68	49.95	53.24	0.65	0.43	0.42
Front. ctx-Prec. gyr.	64.32	11.39	29.2	99.1	22.38	61.71	66.92	0.06	−0.43	0.252
Ins. ctx.	58.78	10.44	40.2	93.8	17.76	56.9	60.67	0.72	0.55	0.273
Temp. ctx.	59.54	9.92	40.5	86.1	16.66	57.74	61.33	0.45	0.05	0.815

**Table 3:** Descriptive statistics, asymmetry, and kurtosis measures of brain parenchyma perfusion in the ACM territory of patients on the ipsilateral side with significant ACI stenosis (54).

Reg.	Mean	SD	Min	Max	CV	CI		Skew	Kurt	*p*-Value
Lent. nuc.	46.84	8.69	30.1	69.9	18.54	44.47	49.21	0.32	0.14	0.978
Front. ctx-Prec. gyr.	54.49	11.94	29.8	86.6	27.42	50.41	58.57	0.25	−0.92	0.793
Ins. ctx.	49.88	10.83	24.5	73	21.72	46.92	52.83	−0.17	−0.66	0.951
Temp. ctx.	52.76	10.93	30.2	70.3	20.72	49.78	55.74	−0.11	−1.13	0.647

**Table 4:** Descriptive statistics, asymmetry, and kurtosis measures of brain parenchyma perfusion in the ACM territory of patients on the contralateral side with non-significant ACI stenosis (54).

Reg.	Mean	SD	Min	Max	CV	CI		Skew	Kurt	*p*-Value
Lent. nuc.	48.16	8.01	30.8	76.9	16.63	45.97	50.35	0.93	2.22	0.188
Front. ctx.-Prec. gyr.	62.96	9.24	42.8	92.9	19.44	59.62	66.3	0.32	−0.56	0.911
Ins. ctx.	51.49	8.86	31.1	65.2	17.2	49.08	53.91	−0.37	−0.91	0.904
Temp. ctx.	57.6	9.35	40.2	77.8	16.23	55.04	60.15	0.04	−0.85	0.604

**Table 5:** The results of the paired-samples *t*-test comparing cerebral blood flow (CBF) across three groups at four brain regions. Group 1 comprises CBF values from control subjects; Group 2 includes CBF values measured ipsilateral to the stenosed artery in patients with significant internal carotid artery (ICA) stenosis; Group 3 encompasses CBF values measured in the hemisphere contralateral to the stenosed artery in the same patient cohort. The table provides the mean CBF values for each group, along with t-values and *p*-values for the comparisons.

Brain Region	Comparison	Mean Group 1	Mean Group 2	t-Value	*p*-Value
Lentiform Nucleus	1 vs. 2	51.595	46.838	3.23	0.001
Lentiform Nucleus	1 vs. 3	51.595	48.161	2.383	0.018
Precentral Gyrus	1 vs. 2	64.315	54.489	4.117	<0.001
Precentral Gyrus	2 vs. 3	54.489	62.957	3.221	0.002
Insular Cortex	1 vs. 2	58.784	49.876	5.147	<0.001
Insular Cortex	1 vs. 3	58.784	51.494	4.458	<0.001
Temporal Cortex	1 vs. 2	59.538	52.761	4.039	0.001
Temporal Cortex	2 vs. 3	52.761	57.596	2.471	0.015

**Table 6:** Characteristics of cerebral blood flow (CBF) in three groups: ipsilateral, contralateral, and healthy, across four examined brain regions. The table also includes the results of the Student’s *t*-test, with asterisks indicating statistically significant differences. The ** (double asterisk) next to the highest values signifies a significantly preserved or relatively higher CBF compared to both the decreased and moderate groups. The * (single asterisk) denotes a significant difference compared to the group with lower values. If * appears next to the moderate group, it indicates a significantly higher perfusion relative to the decreased group.

Brain Region	Control	Ipsilateral	Contralateral	Contribution (%)
Insular Cortex	Higher **	Decreased	Intermediate	64.684
Precentral Gyrus	Higher *	Decreased	Intermediate *	18.216
Temporal Cortex	Higher *	Decreased	Intermediate *	10.781
Lentiform Nucleus	Higher **	Decreased	Intermediate	6.32
Number of Subjects	78/120	32/54	37/54	
Percentage (%)	65.00	59.26	68.52	

## Data Availability

The human data supporting the findings of this study are not publicly available due to ethical considerations, including participant privacy and data sensitivity. However, the data may be provided by the corresponding author upon request, subject to appropriate institutional and ethical approvals.

## Abbreviations

The following abbreviations are used in this manuscript:

ASL	Arterial Spin Labeling
CBF	Cerebral Blood Flow
TIA	Transient Ischemic Attack
ICA	Internal Carotid Artery
MCA	Middle Cerebral Artery
ACoA	Anterior Communicating Artery
PCoA	Posterior Communicating Artery
MRI	Magnetic Resonance Imaging
pCASL	Pseudo-Continuous Arterial Spin Labeling
PET	Positron Emission Tomography
[^15^O]H_2_O	Oxygen-15-labeled water
DM	Diabetes Mellitus
HTA	Hypertension
HLP	Hyperlipoproteinemia
DWI	Diffusion-Weighted Imaging
FLAIR	Fluid-Attenuated Inversion Recovery
SWAN	Susceptibility-Weighted Angiography
BRAVO	Brain Volume Imaging
3D TOF	Three-Dimensional Time-of-Flight
ROI	Region of Interest
SNR	Signal-to-Noise Ratio
TR	Repetition Time
TE	Echo Time
LD	Labeling Duration
PLD	Post-Labeling Delay
NSA/NEX	Number of Signal Averages/Excitation
MANOVA	Multivariate Analysis of Variance
ANOVA	Analysis of Variance
DA	Discriminant Analysis
CV	Coefficient of Variation
CI	Confidence Interval
Skew	Skewness
Kurt	Kurtosis
CEA	Carotid Endarterectomy
CAS	Carotid Artery Stenting
CVR	Cerebrovascular Reserve
OEF	Oxygen Extraction Fraction
TCD	Transcranial Doppler
ESVS	European Society for Vascular Surgery
